# Differential analysis of the quality and soil microhabitat of *Epimedium koreanum* Nakai under different cultivation methods

**DOI:** 10.3389/fmicb.2025.1556173

**Published:** 2025-05-22

**Authors:** Yonggang Zhang, Huiling Hou, Yanxia Li, Kexin Li, Hongmei Lin

**Affiliations:** ^1^College of Chinese Medicinal Materials, Jilin Agricultural University, Changchun, China; ^2^Cultivation Base of State Key Laboratory for Ecological Restoration and Ecosystem Management of Jilin Province, Ministry of Science and Technology, Changchun, China

**Keywords:** *Epimedium koreanum* Nakai, rhizosphere microbial, pharmacodynamic components, soil properties, soil enzyme activity

## Abstract

**Introduction:**

*Epimedium koreanum* is a traditional Chinese tonic medicine obtained from the wild and cultivation, and its pharmacodynamic composition differs depending on the artificial cultivation method. Rhizosphere microorganisms influence the growth and active component accumulation of medicinal plants; however, the detailed composition, diversity, and connections to soil properties and medicinal herb active components in *E. koreanum* remain under-researched.

**Methods:**

Illumina NovaSeq technology was used to study the differences in rhizosphere microbial diversity and composition and pharmacodynamic constituents among cultivation methods, including wild tending (WT), bionic cultivated in forest (FP), and simulated habitat cultivation (SC).

**Results:**

Compared with estimates for WT, SC and FP resulted in higher contents of active components in *E. koreanum*. This cultivation method improves the soil environment by increasing the soil pH, AK, TK, and AP contents. pH, TK, and AP were key factors affecting the bacterial community, while TN, AN, and SOM had significant impacts on the fungal community. Further analyses indicated that the active components of *E. koreanum* are positively correlated with the abundance of microorganisms, such as *Bacillus* and *Humicola*. These microbial communities were significantly enriched in the rhizosphere of FP. In addition, the rhizospheres of FP and WT were enriched with microbial taxa related to plant stress resistance, indicating that different cultivation methods have differential regulatory effects on the plant rhizosphere environment. The proposed FP cultivation method focuses primarily on nitrogen reduction and phosphorus-potassium enhancement along with microbial regulation to synergistically improve both medicinal quality and ecological adaptability.

**Conclusion:**

Soil physicochemical properties and enzymatic activity under different cultivation methods affect soil microbial diversity and composition, thereby impacting plant growth and the synthesis of key components. This work provides a theoretical basis for the scientific and effective cultivation of high-quality *E. koreanum*.

## 1 Introduction

Woodlands and grasslands are natural breeding grounds for many medicinal plants. Currently, in China, under the premise of maintaining ecological security and utilizing the advantages of forest and grassland resources, the optimization of ecological cultivation methods to produce safe, efficient, green, and organic high-quality Chinese herbal medicines is necessary to meet demand. China is the modern center of the geographic distribution of Epimedium in the family Berberidaceae. *Epimedium koreanum* Nakai is the only species in the genus Epimedium distributed in northeastern China, northern Korea, and Japan, and grows naturally in the understory or thickets. *E. koreanum*, as one of the basal elements of the traditional Chinese medicine Herba Epimedii, is included in the Pharmacopoeia of the People’s Republic of China. The dried leaves are used in medicine and have various functions, including tonifying kidney yang, strengthening muscles and bones, and dispelling wind-dampness. Icariin, epimedin and flavonoids are important indicators for evaluating the quality of medicinal materials (Chinese Pharmacopoeia Commission, 2020). In modern medical clinical practice, it has also applied it to the treatment of Alzheimer’s disease (Wang R. et al., 2023), anti-tumor purposes (Sun et al., 2021) and other diseases. Over 90% of the medicinal material supply of *E. koreanum* relies on wild resources. However, the destruction of its natural habitats, continuously growing market demand, and excessive harvesting have led to a sharp decline in wild populations (Li Z. et al., 2024). Therefore, it is necessary to develop appropriate cultivation methods, including artificial cultivation, to address the gap between raw material supply and demand and to protect and rationally utilize wild *E. koreanum* resources.

With the increasing market demand for medicinal plants, many countries have listed medicinal plants as important economic crops. Soil is the basis of medicinal plant cultivation, and there are extremely complex interactions between medicinal plants and soil microorganisms. Specific environmental conditions create specific soil microbiological systems, which have an impact the herbal medicine quality. The plant root system involves the aggregation of microbial communities in the soil micro-ecosystem, providing important ecological niches for interactions between plant roots and microorganisms (Trivedi et al., 2020). The structure and dynamics of rhizosphere microbial communities are important drivers for maintaining a soil micro-ecological balance, measuring soil quality, and maintaining soil fertility and plant stress tolerance (Fitzpatrick et al., 2020; Zhou et al., 2021), and improving the utilization of mineral elements in the soil. Numerous studies have pointed out that the active flora in the rhizosphere soil can greatly promote the enrichment of nutrients in the soil, which has important implications for the production of high-quality medicinal herbs (Feng et al., 2021; Su et al., 2023). The application of the rhizosphere-promoting bacterium *Azotobacter chroococcum* and *Azospirillum brasilense* alleviated the adverse effects of drought on *Mentha pulegium* L. significantly and increased the contents of phenolic compounds, flavonoid, and essential oil (Asghari et al., 2020). Zhang et al. (2021) found that *Streptomyces netropsis* WLXQSS-4 isolated from the rhizosphere of *Clematis manshurica* Rupr. contains genes related to the biosynthesis of alloaureothin. *Burkholderia* promotes the synthesis of indigo, a main active ingredient of *Baphicacanthus cusia* (NeeS) Bremek (Zeng et al., 2018). *Bryobacter* and *Candidatus Solibacter* can indirectly increase the polysaccharide content of *Polygonatum cyrtonema* Hua (Liu J. et al., 2024). Similar studies have emphasized the ability of rhizosphere microbiota, especially several key taxa, to increase the ginsenoside content in *Panax ginseng* as well as polysaccharides and flavonoids in *Dendrobium officinale* (Dong et al., 2019; Zhou et al., 2025).

Despite research on ecological cultivation, such as wild-tending and ecological planting of *E. koreanum*, the weak seed propagation ability of the species limits the supply of seeds and seedlings, thereby limiting artificial cultivation (Dai et al., 2022; Yan et al., 2021; Zhang et al., 2024). The relationships between the rhizosphere microbial composition, diversity of *E. koreanum* under different ecological cultivation methods, and soil properties and pharmacodynamic components have not been clearly established. Therefore, this study utilized Illumina NovaSeq technology to investigate the diversity and composition of the rhizosphere soil microbial community of *E. koreanum*, in addition to analyses of soil physicochemical properties, enzyme activity, pharmacodynamic components, and correlations between these parameters. The results of this study not only deepen our understanding of the rhizosphere microorganisms of *E. koreanum* but also provide a valuable scientific reference for rational planting and soil cultivation.

## 2 Materials and methods

### 2.1 Sample collection and pre-processing

Based on previous research on *E. koreanum*, late May (the full bloom period) and late August (the late post-fruit growth period) are the key periods for the synthesis and accumulation of pharmacodynamic components (Tang et al., 2007). Plant and rhizosphere soil samples in this study were collected in late May and late August of 2023. Samples of *E. koreanum* from bionic cultivation in the forest (bionic cultivation in a *Larix* forest, FP) and cultivation under a simulated habitat (cultivation at logging sites covered with shade nets, SC) were collected from the Epimedium planting base in Tonghua County, Jilin Province, China (125°28.7′E, 41°39.44′N). Wild *E. koreanum* samples collected from an artificial closure area near the base (WT). All cultivation methods avoided human interference and no exogenous fertilizers were applied. The climatic conditions were the same at each collection site.

The 5-point sampling method was used to collect *E. koreanum* plants and rhizosphere soil. The whole plant was dug out with soil, the loose soil and impurities were shaken from the roots, and the rhizosphere soil of the fibrous roots was collected using a sterile brush. All samples were placed in a low-temperature storage box and quickly brought back to the laboratory. One soil sample was encapsulated in a sterile EP tube and stored at −80°C for DNA extraction and high-throughput sequencing; one soil sample was dried naturally and sieved through a 20-mesh sieve for the determination of physicochemical properties and enzyme activities. Leaf samples were naturally dried, powdered, and sieved for the determination of major flavonoid contents.

### 2.2 Determination of the content of the main flavonoid components

Determination was performed with reference to the “Content Determination” method under “Epimedium” of the 2020 edition of Chinese Pharmacopoeia (Chinese Pharmacopoeia Commission, 2020). Determination of icariin, epimedin A, epimedin B, and epimedin C by high-performance liquid chromatography (Agilent 1260 HPLC, Agilent). Meanwhile, the content of total flavonoids was determined using UV spectrophotometry.

### 2.3 Soil physicochemical properties and enzyme activity tests

Determination of physicochemical properties and enzymatic activities of rhizosphere soils followed relevant standard soil test methods (Bao, 2000). The soil pH value was determined using an international standard 1:5 soil-water ratio while using an ion meter (MP521, Sanxin Instrument Factory, Shanghai, China); the soil organic matter (SOM) content was determined using potassium dichromate oxidation-colorimetry method with water hydration; the total nitrogen (TN) was determined using Kjeldahl nitrogen determination (K9860, Haineng Scientific Instrument Co., Ltd., Shandong, China); determination of ammonia nitrogen (AN) by the alkaline diffusion method; the total phosphorus (TP) was determined using the NaOH melt-molybdenum-antimony anticolorimetric method; determine the available phosphorus (AP) content by the 1/2H_2_SO_4_ method; the available potassium (AK) and total potassium (TK) were determined using flame atomic absorption spectrophotometry (AA-6300, Shimadzu Corporation, Japan). Soil saccharase (SSC) activity was determined by colorimetric assay with 3,5-dinitrosalicylic acid; soil urease (SUE) activity was determined by indophenol blue colorimetric assay; soil acid phosphatase (SACP) was determined by colorimetric assay with sodium benzene diphosphate (Solarbio, BC0145); soil catalase (SCAT) was determined by ultraviolet spectrophotometry (Solarbio, BC0105).

### 2.4 DNA extraction, amplification and sequencing techniques

Soil DNA extraction was performed using the HiPure Soil DNA Extraction Kit (Magen, Guangzhou, China). The purity and concentration of DNA were detected by using a NanoDrop micro-volume spectrophotometer and agarose gel electrophoresis. Amplification of the highly variable V3–V4 region of the bacterial 16SrRNA gene using specific primers 341F (5′-CCTACGGGNGGCWGCAG-3′) and 806R (5′-GGACTACHVGGGGTATCTAAT-3′). The transcribed spacer region of the fungal ITS gene was then amplified by PCR using primers ITS3 KYO2 (5′-GATGAAGAACGYAGYRAA-3′) and ITS4 (5′-TCCTCCGCTTATTGATATATGC-3′). PCR amplification conditions: pre-denaturation at 95°C for 5 min; 30 cycles (denaturation at 95°C for 1 min, annealing at 60°C for 1 min, and extension at 72°C for 1 min); final extension at 72°C for 7 min. Amplification system: 50 μL mixture containing 10 μL of 5 × Q5@ Reaction Buffer, 10 μL of 5 × Q5@ High GC Enhancer, 1.5 μL of 2.5 mM dNTPs, 1.5 μL of each primer (10 μM), 0.2 μL of Q5@ High-Fidelity DNA Polymerase, and 50 ng of template DNA. Related PCR reagents were from New England Biolabs, United States (Supplementary Tables S1, S2). 2% agarose gels were assessed for amplification product quality for integrity. The sequencing library was constructed using the Illumina DNA Prep Kit (Illumina, CA, United States).

Using the NovaSeq 6000 platform, 2 × 250 bp paired-end reads were generated. Filtering, merging, quality control, and organization of raw data were performed to obtain valid data. Chimeric sequences were identified by comparisons against the SILVA (16S, version 138.1) and UNITE (ITS, version 8.3) databases. Taxonomic annotations were performed using RDP annotation software (version 2.2). Soil DNA extraction, amplification, and sequencing were performed by Gene Denovo Corporation (Guangzhou, China).

### 2.5 Data and statistical analysis

Alpha and beta diversity were analyzed with standardized datasets using the vegan package in R and a nonparametric multivariate analysis of variance (Adonis). Differences in microbial diversity and relative abundance at different microbial classification levels between groups were assessed using one-way ANOVA, *t*-tests, or Kruskal–Wallis tests using SPSS software. Mantel’s tests, Pearson’s correlation analyses, and structural equation modeling (SEM) were utilized to explore the relationships between medicinal components, soil properties, and microorganisms. Sequencing data were analyzed using the Omicsmart cloud platform.^1^

## 3 Results

### 3.1 Soil physicochemical properties, enzyme activities, and content of medicinal components

**Figure 1:**
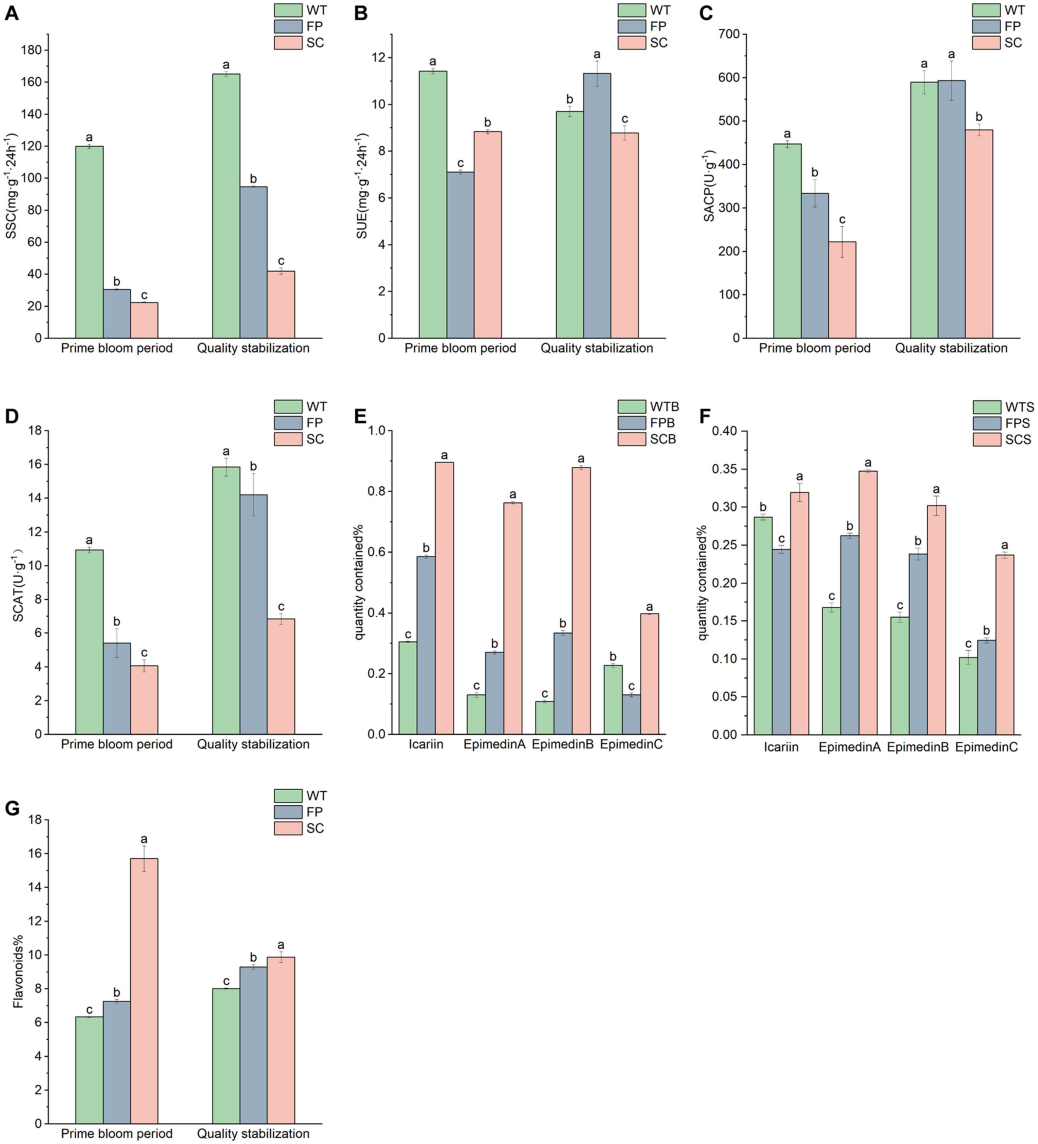
Soil enzyme activities and contents of pharmacodynamic components under different cultivation methods. **(A)** Soil saccharase. **(B)** Soil urease. **(C)** Soil acid phosphatase. **(D)** Soil catalase; full bloom period. **(E)** Late post-fruit nutrient growth period. **(F)** Epimedin A, B, and C contents. **(G)** Total flavonoid content. The letters “a, b and c” are significant markers, and different letters between treatments indicate significant differences at the significant level of 0.05. WT, FP, and SC, respectively, correspond to three cultivation methods. The suffix-B represents the full-bloom stage, and -S represents the post-fruit nutritional growth late stage. For example, WTB represents a sample in the full-bloom stage of wild tending, and WTS represents a sample in the post-fruit nutritional growth late stage of wild tending.

Table 1 shows the physicochemical properties of the soil for different cultivation methods. The soil was generally strongly acidic. The pH value was significantly higher during the late post-fruit nutrient growth period than at the full bloom period. The pH values for FPB were significantly lower than those of WTB and SCB, showing very strong acidity, and the pH of SCS was significantly higher than that of WTS and FPS. The SOM, AN, and TN contents in the rhizosphere soil of SC was significantly lower than that of WT and FP (*p* < 0.05). The differences in the TP content among cultivation methods were not significant. However, at the late nutritional stage of post-fruit growth, AP contents decreased in the order SC > FP > WT and were significantly lower in WT than in FP and SC. WT samples showed a significantly lower AK and TK contents than that in FP and SC, and WTS > WTB, while FPS < FPB, SCS < SCB. In addition, the AP contents were significantly higher in the late stage of post-fruiting nutrient growth than in the full bloom stage for each cultivation method. The SSC, SACP, and SCAT activity generally decreased in the order of WT > FP > SC. SSC, SACP, and SCAT activity was significantly higher during the late post-fruiting nutrient growth stage than at the full bloom period (Figures 1A,C,D). SUE activity was significantly lower in FPB than in WTB and SCB, whereas FP > WT > SC was observed at the later stages of post-fruiting nutrient growth (Figure 1B). The total contents of medicinal components under different cultivation methods for *E. koreanum* followed the order SC > FP > WT (Figures 1E–G). The contents of total flavonoids and total flavonol glycosides (icariin, epimedin A, B, and C sum) of WTB, FPB, and SCB, respectively, were 6.33, 0.78%; 7.26, 1.32%; 15.71, 2.94%. The contents of total flavonoids and total flavonol glycosides of WTS, FPS, and SCS were 8.02, 0.71%; 9.30, 0.87%; 9.88, 1.21%, respectively. All values exceeded the standards reported in Chinese Pharmacopoeia.

**Table 1:** Soil physicochemical properties under different cultivation methods.

Soil indicators	WTB	FPB	SCB	WTS	FPS	SCS
pH	4.61 ± 0.02aB	4.18 ± 0.06bB	4.58 ± 0.11aB	5.20 ± 0.17bA	5.18 ± 0.04bA	5.60 ± 0.01aA
SOM (%)	2.09 ± 0.01aB	2.14 ± 0.02aB	1.53 ± 0.02bB	2.81 ± 0.03aA	2.53 ± 0.03bA	1.87 ± 0.06cA
AN (mg kg^−1^)	354.67 ± 6.17aB	291.67 ± 6.17bB	221.67 ± 6.17cA	476 ± 21.39aA	396.67 ± 6.17bA	228.67 ± 6.17cA
AP (mg kg^−1^)	44.72 ± 0.47cB	60.81 ± 1.12bB	68.79 ± 0.93aA	59.16 ± 0.82cA	87.23 ± 0.94aA	66.09 ± 0.47bA
AK (mg kg^−1^)	364.14 ± 2.68bB	512.9 ± 23.00aA	510.8 ± 32.15aA	397.88 ± 5.67bA	404.77 ± 8.00bB	454.31 ± 10.33aB
TN (g kg^−1^)	5.49 ± 0.01aB	3.92 ± 0.03bB	3.11 ± 0.03cA	6.16 ± 0.07aA	4.78 ± 0.06bA	2.89 ± 0.01cB
TP (g kg^−1^)	1.24 ± 0.03aA	1.29 ± 0.02aA	1.30 ± 0.10aA	0.93 ± 0.07cB	1.18 ± 0.05bB	1.32 ± 0.05aA
TK (g kg^−1^)	7.4 ± 0.22bB	11.7 ± 0.23aA	11.69 ± 0.25aA	9.04 ± 0.65bA	10.45 ± 0.65aB	10.59 ± 0.52aB

Values are means ± standard error. Significant differences are indicated by letters (*p* < 0.05). Lowercase letters denote differences between cultivation modalities, while uppercase letters denote differences between periods. WT, FP, and SC, respectively, correspond to three cultivation methods. The suffix-B represents the full-bloom stage, and -S represents the post-fruit nutritional growth late stage. For example, WTB represents a sample in the full-bloom stage of wild tending, and WTS represents a sample in the post-fruit nutritional growth late stage of wild tending.

### 3.2 Analysis of soil microbial diversity and composition

#### 3.2.1 Analysis of soil microbial community diversity

**Figure 2:**
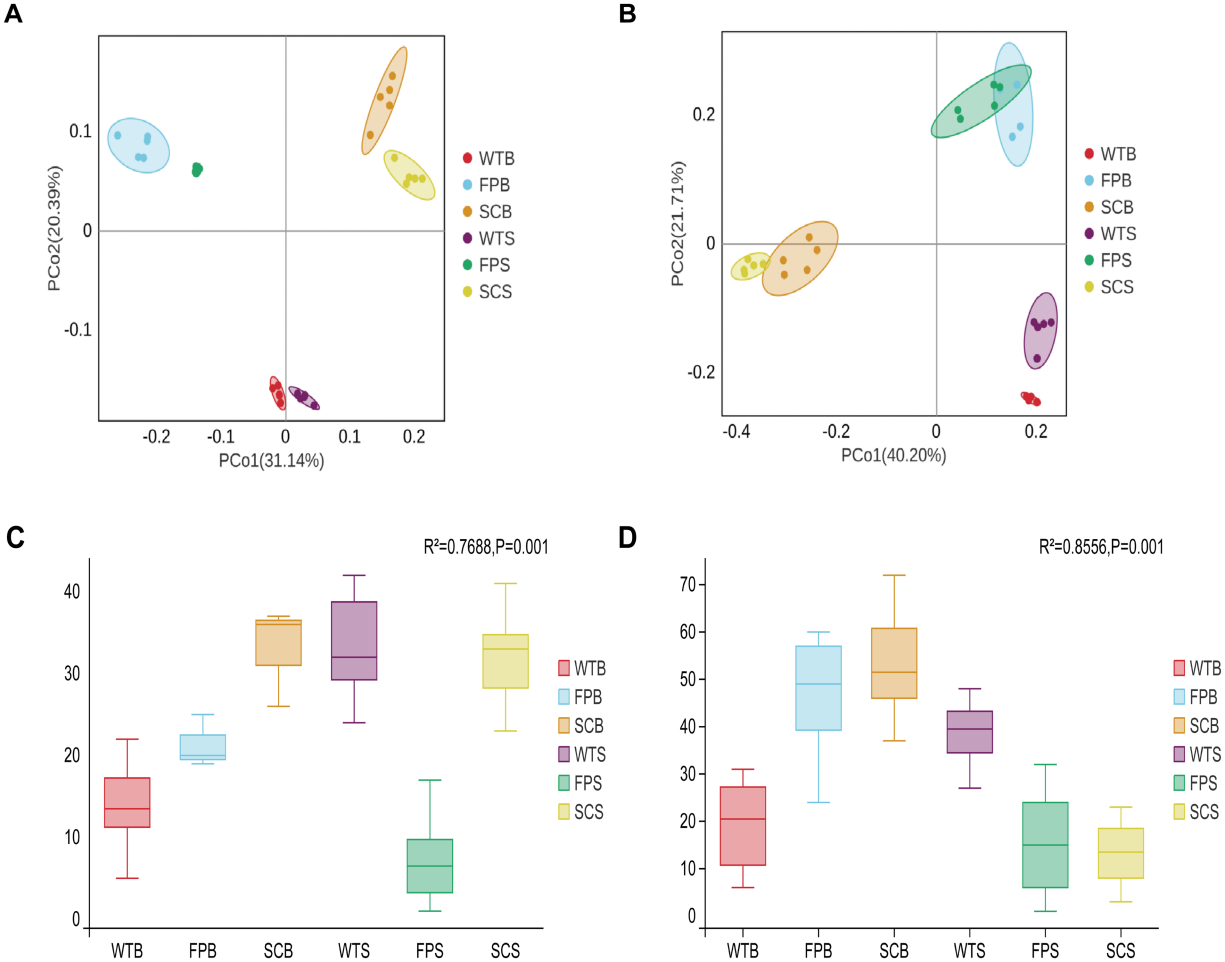
Bacteria **(A)** and fungi **(B)** under different cultivation methods principal coordinate analysis (PCoA). Bacterial **(C)** and fungal **(D)** Adonis (PERMANOVA) box test plots.

A rarefaction curve analysis showed stabilization with increasing sequencing volumes, indicating that the sequencing depth was sufficient to reflect the structure and composition of bacterial and fungal communities in the rhizosphere soil samples under different cultivation methods (Supplementary Figure S1). In bacteria, there were significantly fewer the OTUs, Shannon, and Chao1 index in FP samples than in WT and SC samples. From the full-bloom to post-fruit nutritional growth late stage, the number of the OTUs, Shannon index, and Chao1 index of WT and FP samples increased significantly; however, the general pattern, WT > FP > SC, indicated that the species richness and diversity of rhizosphere bacteria in WT and SC were significantly higher than those in FP. In terms of fungal diversity, there were significantly more OTUs, Shannon, and Chao1 index during the two periods in SC and FP samples than in other samples during the corresponding periods; rhizosphere fungal diversity and abundance were higher in WT and lower in FP and SC (Table 2). Figure 2 shows the principal coordinates analysis (PCoA) results for soil bacteria and fungi, illustrating the differences in the composition of the rhizosphere soil microbial community between cultivation methods. In an Adonis (PERMANOVA) analysis, as summarized in Figure 2, the *R*^2^ value was close to 1, indicating that the block effect has a high explanatory power for sample variation. As shown Figure 2A, the first and second coordinate axes explained 31.14 and 20.39% of the variance in bacterial community composition, respectively. The rhizosphere soil bacterial communities of WT, FP, and SC were clearly separated (*R*^2^ = 0.7668, *p* < 0.001) and could be divided into three distinct groups (Figure 2C). In the PCoA of fungal communities, PCoA1 accounted for 40.20% of the total variance, and PCoA2 accounted for 21.71%. The rhizosphere soil fungal communities under three cultivation methods showed significant differences (*R*^2^ = 0.8556, *p* < 0.001), indicating that different cultivation methods had a significant impact on the structure of soil fungal communities (Figures 2B,D). Therefore, rhizosphere microbial communities of *E. koreanum* cultivated using different methods have different structures.

**Table 2:** Microbial diversity index under different cultivation methods.

/	Diversity index	WTB	FPB	SCB	WTS	FPS	SCS
Bacteria	OTUs	4097.50 ± 88.62a	3350.50 ± 91.15b	4196.50 ± 92.01a	4566.00 ± 101.36a	3605.75 ± 60.53b	4,519 ± 97.27a
	Shannon	9.75 ± 0.06b	9.26 ± 0.18c	10.14 ± 0.06a	10.30 ± 0.02a	9.56 ± 0.02b	10.01 ± 0.32a
	Chao1	4348.13 ± 82.35a	3564.07 ± 125.52b	4460.89 ± 136.11a	4851.8 ± 182.32a	3828.85 ± 87.48b	4503.29 ± 345.86a
Fungi	OTUs	2194.5 ± 46.94a	1913 ± 140.77b	1934.5 ± 31.25b	2,295 ± 46.22a	1882.25 ± 73.56b	1855.75 ± 34.85b
	Shannon	7.34 ± 0.09a	7.22 ± 0.15a	6.76 ± 0.36b	7.64 ± 0.09a	7.07 ± 0.04b	6.52 ± 0.13c
	Chao1	2240.40 ± 55.13a	2044.07 ± 134.65b	1974.69 ± 75.46b	2330.87 ± 61.55a	1946.21 ± 59.94b	1863.66 ± 79.53b

Lowercase letters indicate differences between different cultivation methods (*p* < 0.05).

#### 3.2.2 Soil microbial community composition

**Figure 3:**
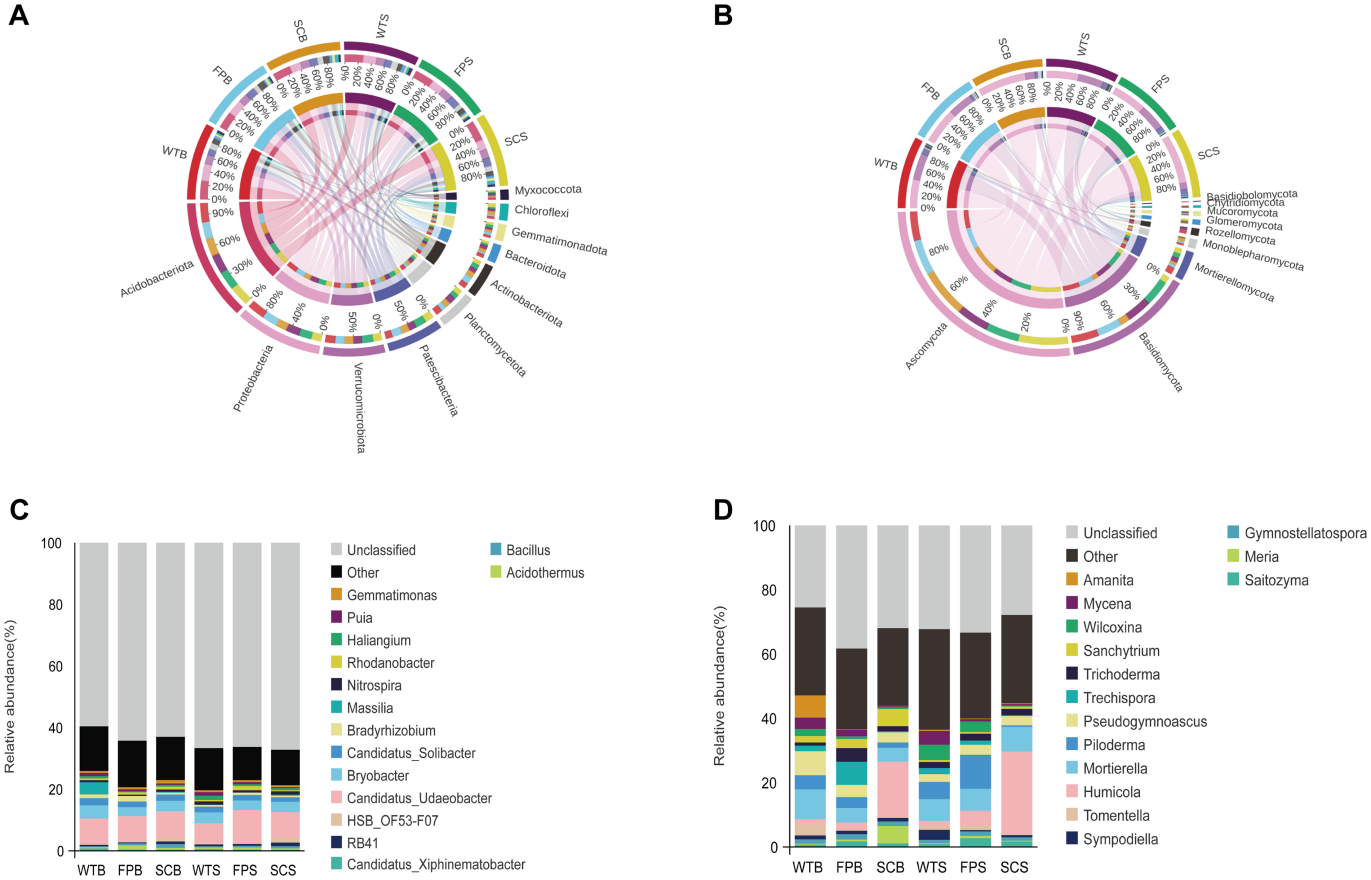
Composition at the level of **(A,B)** phylum. (**C,D**) Genus under different cultivation methods. **(A,C)** Bacteria. **(B,D)** fungi.

To analyze the composition of the soil bacterial and fungal communities in-depth, the top 10 dominant phylum in terms of relative abundance as well as the top 15 dominant genera were selected for further analyses (Figure 3). *Acidobacteriota* was dominant in most of the samples with a relative abundance of 22.99–26.08%. *Proteobacteria* (12.90–21.85%), *Verrucomicrobiota* (10.65–12.08%), and *Patescibacteria* (8.04–13.80%) were relatively abundant bacterial phylum. However, the relative abundance of soil bacterial phyla under different cultivation methods differed significantly (Figure 3A). For example, in WT samples, the relative abundance of *Proteobacteria* was consistently higher than that of SC and FP samples, whereas the relative abundance of *Patescibacteria* was consistently lower in WT than in SC and FP. *Ascomycota* and *Basidiomycota* were the most abundant fungal phylum, with relative abundances above 81% (Figure 3B). However, the relative abundances of soil fungal phyla differed significantly among cultivation methods. The relative abundances of *Patescibacteria*, *Actinobacteriota*, *Gemmatimonadota*, and other taxa in the FP rhizosphere soil were significantly higher than those in WT and SC. The relative abundance of *Chloroflexi* and *Ascomycota* in SC rhizosphere soil was significantly higher than that in WT and FP, while the relative abundance of *Basidiomycota* was lower. Compared with those in the rhizosphere of WT, the relative abundances of *Proteobacteria*, *Mortierellomycota*, and other taxa in the rhizosphere soils of FP and SC were significantly lower.

At the genus level, the dominant bacterium *Candidatus Udaeobacter* showed significant differences during the later stage of post-harvest vegetative growth, decreasing in the order FP > SC > WT (Figure 3C). The abundances of *Rhodanobacter* and *Acidothermus* in the rhizosphere soil of FP were significantly higher than those in WT and SC. The abundances of *Nitrospira*, *HSB OF53-F07*, *RB41* and *Bacillus* in the rhizosphere soil of SC were significantly higher than those in WT and FP. Among fungi, *Humicola* was the dominant genus in the SC rhizosphere and had a significantly higher abundance in SC than in WT and FP. The abundance of *Mortierella* in the rhizosphere soils of WTB and SCS was significantly higher than those in other samples. The abundance of *Saitozyma* in the rhizosphere of FP was significantly higher than that in WT and SC. In addition, fungi such as *Trechispora*, *Piloderma*, and *Amanita* were significantly more abundant in FP than in other treatments. Other fungi, such as *Wilcoxina* and *Mycena* in the soil of WT and *Mortierella* in the rhizosphere soil of WTB, showed a significantly higher abundance than those in other treatments.

#### 3.2.3 Analysis of soil microbial indicator species

**Figure 4:**
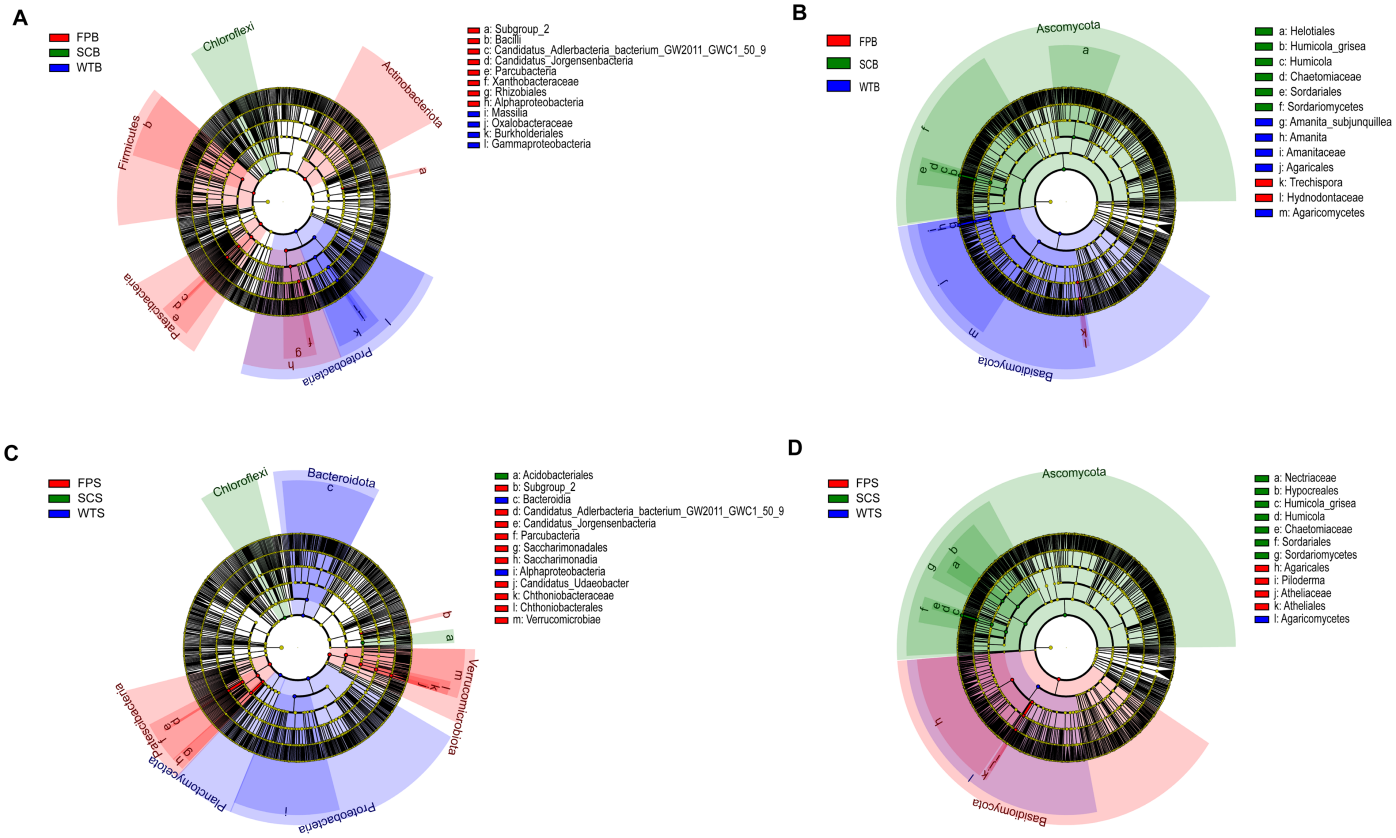
Linear discriminant analysis (LEfSe) plots of different cultivation methods **(A,C)** bacterial and **(B,D)** fungal. Bacteria: LDA ≥4.0; Fungi: LDA ≥4.5. The dendrograms were categorized at the level from phylum to genus. Nodes of different colors indicate microorganisms that were significantly enriched in the corresponding group and had a significant effect on the differences between groups (*p* < 0.05). Light yellow nodes indicate microorganisms that were not significantly different in any of the different groups (*p* > 0.05).

LEfSe (linear discriminant analysis effect size) indicated differences in high-dimensional biomarker taxa in soil bacterial and fungal communities under different cultivation practices (Figure 4; Supplementary Figure S2). The main biomarkers of WT included bacteria such as *Massilia* and *Burkholderiale* as well as fungi such as *Amanitaceae* and *Agaricomycetes*. The main biomarkers of FP included bacteria such as *Subgroup 2*, *Rhizobiales*, and *Bacilli* and fungi such as *Hydnodontaceae*, *Piloderma*, *Trechispora*. The main biomarkers of SC included bacteria such as *Chloroflexi* and *Acidobacteriales* as well as fungi such as *Humicola* and *Helotiales*. The number of differential markers in bacterial communities was higher during the late post-fruiting nutrient growth period relative to the bloom period, while fungal biomarkers showed a decrease.

### 3.3 Relationships among soil physicochemical properties, enzyme activities, and microbial communities

**Figure 5:**
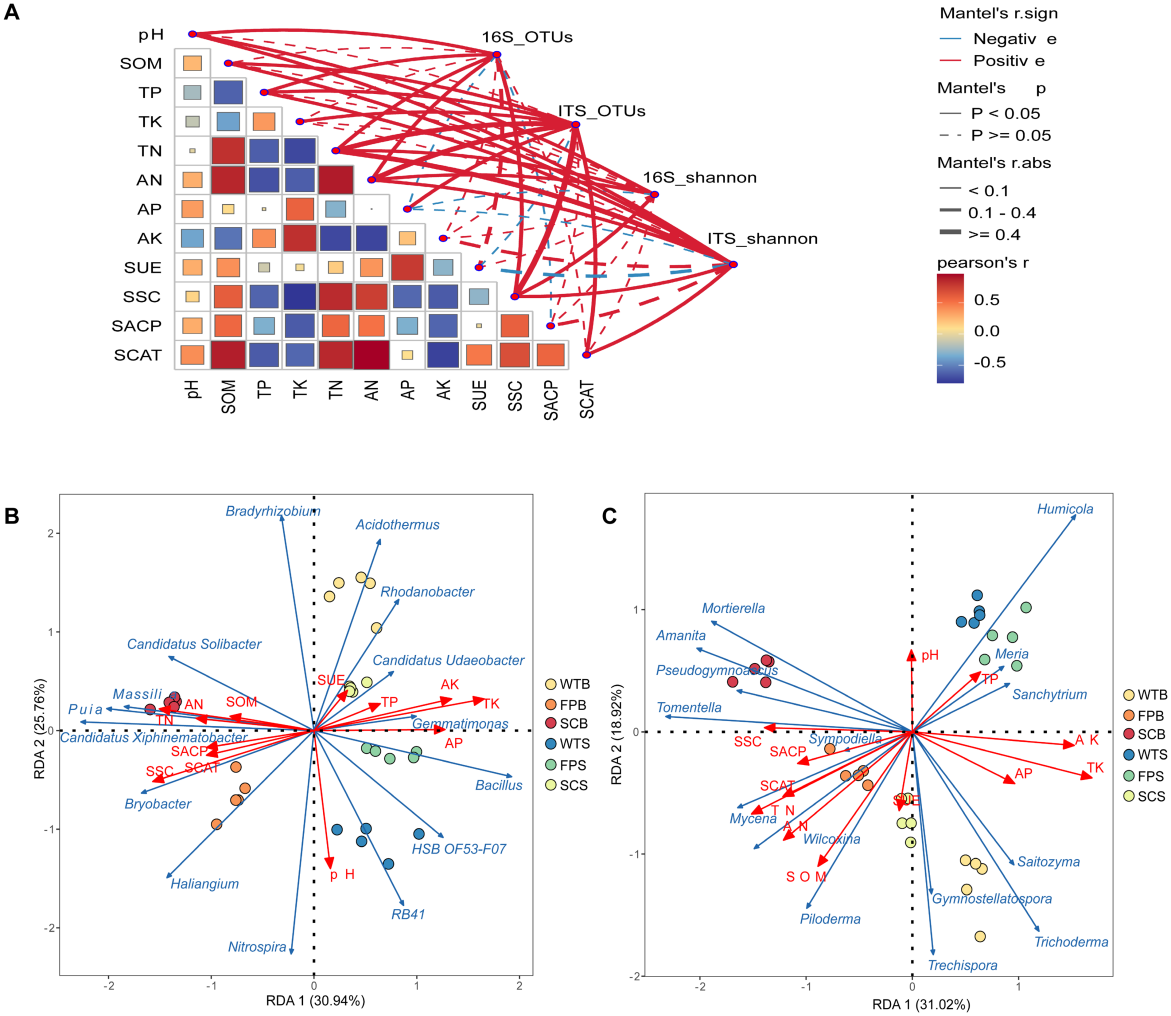
Soil properties and microbial community composition Mantel analysis **(A)**, line width corresponds to Mantel’s *r*-value, color represents significance, Pearson’s correlation coefficients are shown by line color gradient; soil properties with soil bacterial **(B)** and fungal **(C)** RDA analyses, with red arrows indicating the relative position of their physicochemical properties and enzyme activities on the horizontal plane. Blue arrows indicate the distribution of species at the genus level, with longer arrows indicating a greater impact on the species. The strength of the correlation varies when the angle between the arrow and the sorting axis is different. The smaller the angle, the stronger the correlation, and the longer the length of the arrow, the greater the influence of environmental factors.

Using Mantel analysis, found that the diversity of rhizosphere microbial species composition was significantly correlated with soil physicochemical properties (Figure 5A). pH, TK, AP, and SSC had highly significant effects (*p* < 0.01) on bacterial communities, while TN, AN, SOM, and SSC had highly significant effects on fungal communities (*p* < 0.001). To further explore the response of the microbial community to environmental factors, are redundancy analysis (RDA) of the dominant genera in the top 15 for each of the bacterial and fungal abundances with soil properties and enzyme activities was performed. The results showed that the RDA1 and RDA2 axes explained a total of 56.70% of the variance in the composition of soil bacterial communities and 49.94% of the variance in the composition of fungal communities, clarifying the main factors that had a significant effect on the presence of bacterial and fungal communities (Figures 5B,C). *Nitrospira* and *RB41* showed a highly significant positive correlation with pH (*p* < 0.01). *HSB OF53-F07*, *Bacillus*, *Gemmatimona*, and other bacteria, and *Humicola*, *Meria*, and *Sanchytrum* fungi were significantly and positively correlated with AP, AK, and TK contents, in addition, *Candidatus Udaeobacter* was also significantly and positively correlated with AP, TP, and SUE contents (*p* < 0.05). *Bryobacter*, *Puia*, *Piloderma*, *Candidatus Xiphinematobacter*, *Candidatus Solibacter*, *Bradyrhizobium*, *Wilcoxina*, and *Massilia* were significantly and positively correlated (*p* < 0.05) with soil AN, SOM, SSC, SACP, and SCAT content.

### 3.4 Relationship of medicinal components to soil properties, enzyme activities, and microbial communities

**Figure 6:**
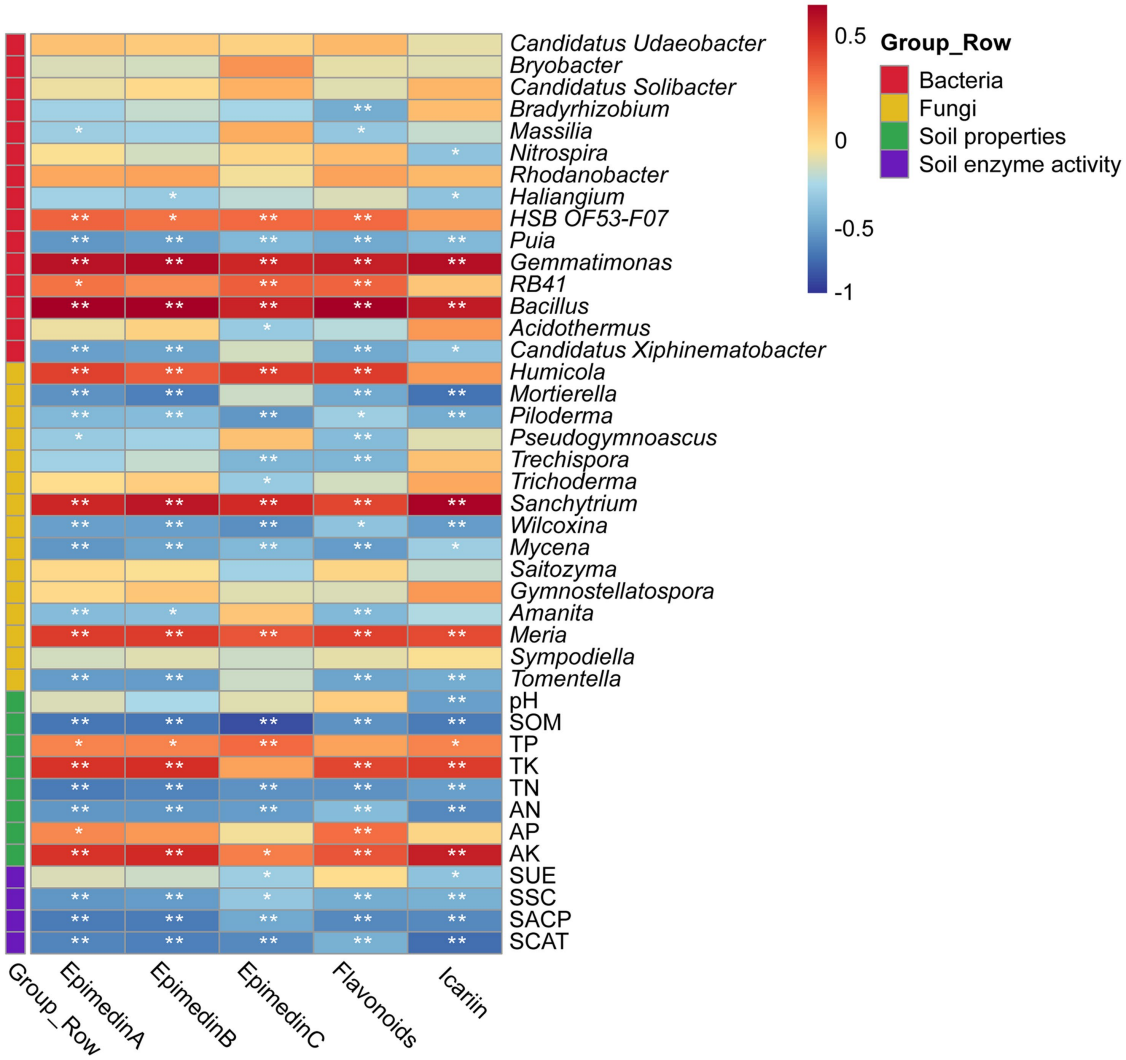
Heat map of correlation between the pharmacodynamic components of *E. koreanum* and soil environmental factors, bacteria and fungi genus levels. “*” Indicates a significant correlation at the 0.05 level, “**” indicates a highly significant correlation at the 0.01 level.

**Figure 7:**
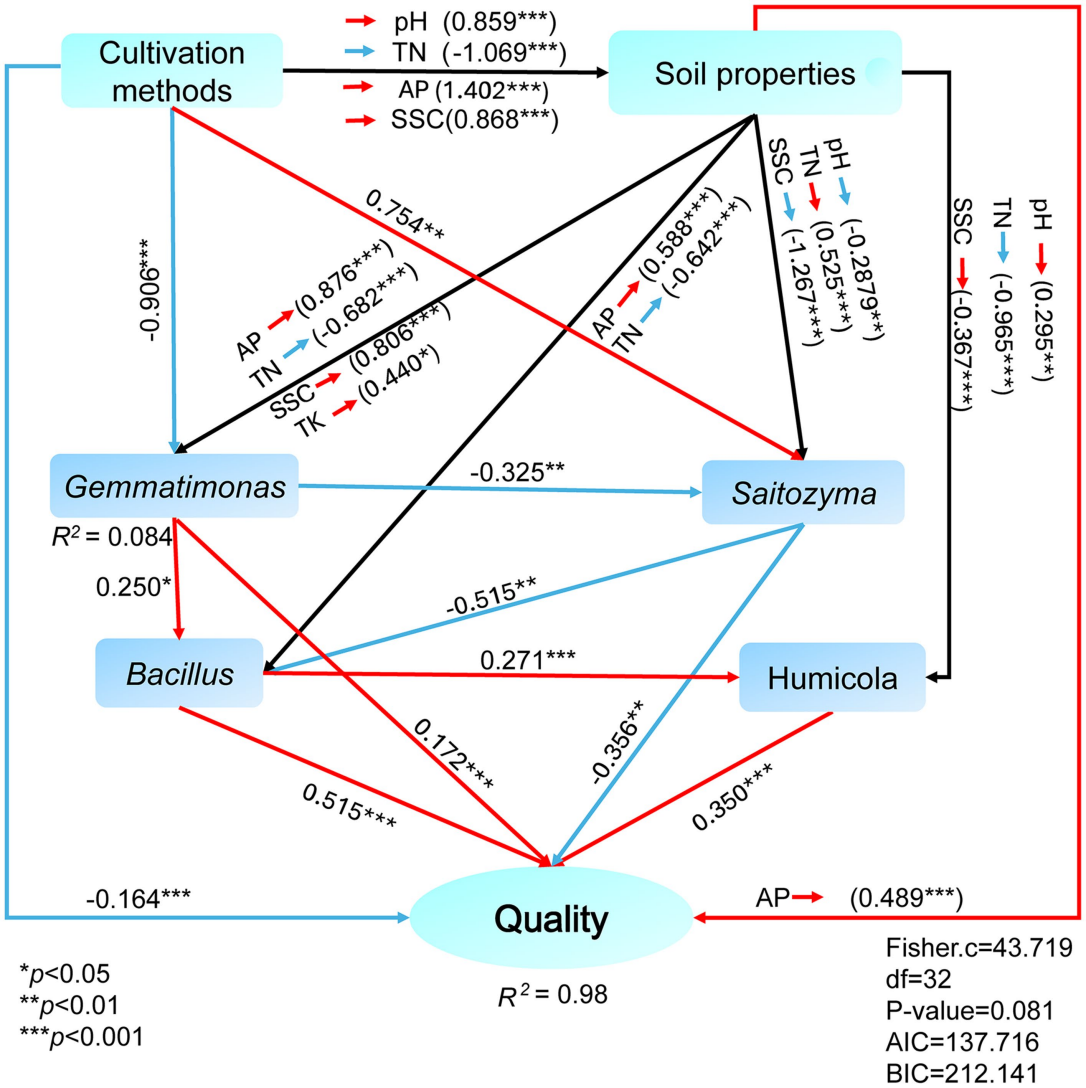
SEM analysis of the correlation between active components of *E. koreanum*, soil environmental factors, and characteristic microorganisms. The red lines represent positive correlations, the blue lines represent negative correlations, and the black lines represent both positive and negative correlations.

Based on Pearson correlation coefficients, there were correlations between the medicinal components of *E*. *koreanum* and the nature of the rhizosphere soil, enzyme activity, and abundances of bacterial and fungal genera under different cultivation methods (Figure 6). The flavonol glycoside and total flavonoid content of *E. koreanum* were extremely significantly positively correlated with AK, TK, TP, *Bacillus*, Gemmatimonas, *Humicola*, *Sanchytrium* (*p* < 0.01). *RB41* and *HSB OF53-F07* was significantly positively correlated with total flavonoids and epimedin content. The above pharmacologically active components were extremely significantly negatively correlated with soil SOM, TN, AN and most fungal genera, such as *Puia*, *Piloderma*, *Wilcoxina*, and *Tomentella* (*p* < 0.01). The influence of characteristic microorganisms on the quality of *E. koreanum* was further explored through SEM (Figure 7). The overall goodness-of-fit of the model was significant (*R*^2^ = 0.98), indicating that the three-level path of cultivation method-soil-microorganisms can explain 98% of the variation in medicinal material quality. *Gemmatimonas*, *Bacillus*, and *Humicola* had a significant impact on the quality of *E. koreanum*. Overall, AP and *Bacillus* had the greatest impact on quality (standardized total coefficients is 0.515, 0.489). TK and SSC was also positively correlated with the abundance of *Gemmatimonas*. The significance of the critical pathway (such as AP → Bacillus → quality) further supports the rationality of the “soil property changes-microbial colonization-component accumulation” process.

### 3.5 Prediction of soil microbial community function

**Figure 8:**
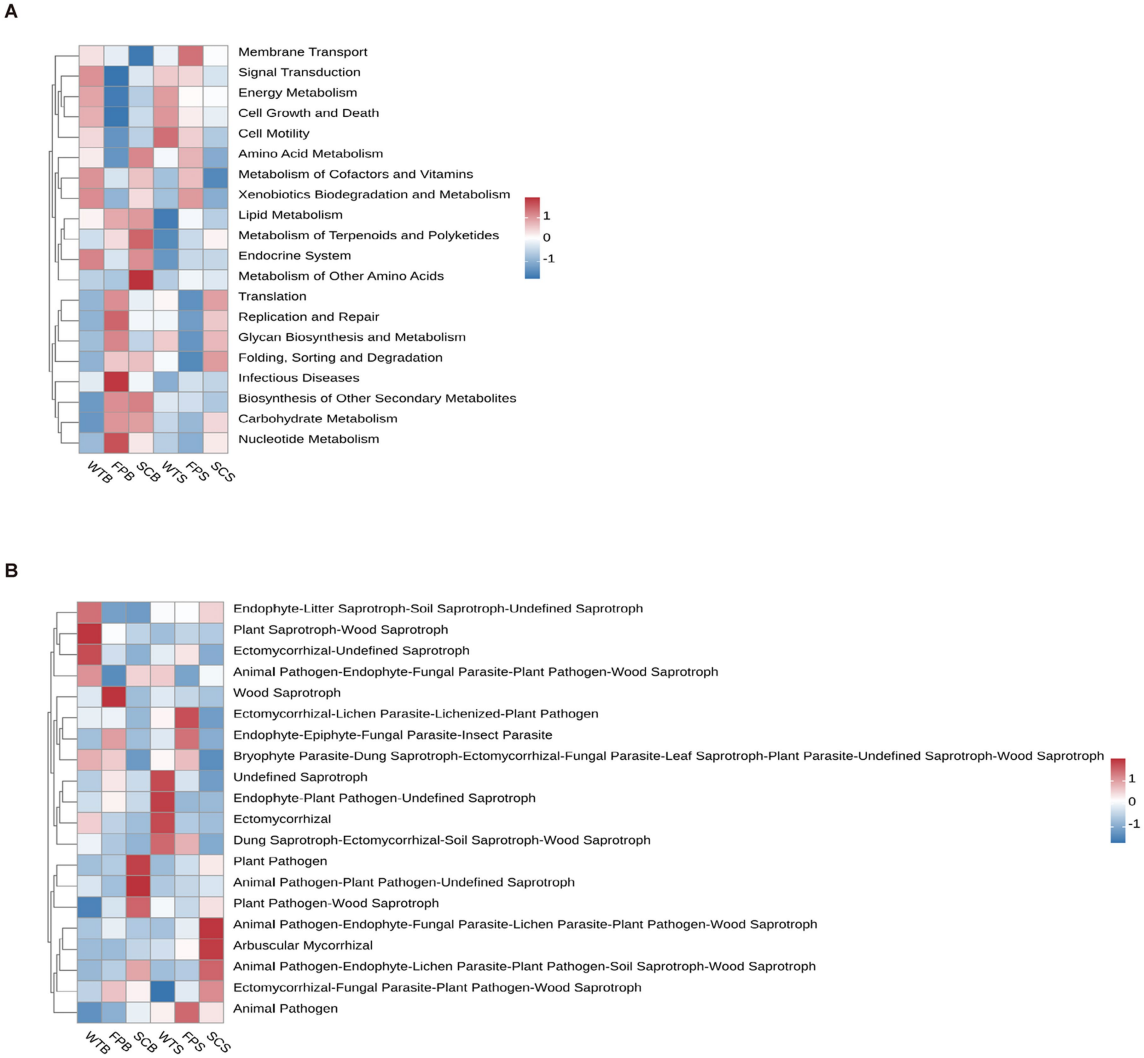
Prediction of functional genes of soil bacterial metabolic pathways KEGG **(A)**. Prediction of functional genes of soil fungal metabolic pathways **(B)**.

The function of the soil bacterial community based on KEGG metabolic pathways during mass stabilization was analyzed using the TAX4Fun prediction method (Figure 8A). Cell motility and endocrine system-related bacterial communities were more enriched in WT than in FP and SC. Membrane transport-related communities in FP were also characterized by significant enrichment. In SC, the abundance of metabolism of other amino acids—related groups was higher than that in WT. Of note, the functional groups did not show significant differences among the three cultivation methods (*p* > 0.05).

A predictive analysis of fungal functions using FUNGuild showed that fungal taxa with unknown functions were dominant and differed significantly in abundance between cultivation methods. The abundances of pathotroph-saprotroph-symbiotroph and saprotroph-symbiotroph fungi were significantly lower in SC than in WT, with decreases reaching 59.25 and 60.59% at the full bloom period, respectively, and 39.75 and 29.93% in the later stages of post-fruit growth and nutrition (Table 3). The abundance of pathotroph-saprotroph in SC was significantly higher than that in WT and was significantly lower during the later stages of post-fruiting nutrient growth. The relative abundance of saprotroph in FP increased by 20.79% at full bloom and decreased by 21.02% at the later stages of post-fruit growth and nutrition compared with those in WT. The relative abundance of bryophytes, endophyte-litter saprotroph-soil saprotroph-undefined saprotropin in WT rhizosphere soil was significantly higher than that in SC. Wood saprotrophs had a significantly higher relative abundance in FP than in WT and FP (Figure 8B).

**Table 3:** Relative abundance of fungal trophic types.

Trophic types	WTB	FPB	SCB	WTS	FPS	SCS
Pathotroph	3.04 ± 0.26c	3.88 ± 0.72b	5.94 ± 0.72a	3.27 ± 0.38c	4.7 ± 0.13b	5.27 ± 0.09a
Pathotroph-saprotroph	2.3 ± 0.10b	2.38 ± 0.48b	3.73 ± 0.10a	2.24 ± 0.25b	1.99 ± 0.10c	2.42 ± 0.09a
Pathotroph-saprotroph-symbiotroph	21.07 ± 0.53a	21.1 ± 2.78a	10.52 ± 0.98c	19.05 ± 1.27b	26.95 ± 0.80a	11.3 ± 0.11c
Pathotroph-symbiotroph	0.81 ± 0.05c	1.66 ± 0.32a	1.15 ± 0.22b	1.17 ± 0.12b	1.91 ± 0.03a	1.04 ± 0.07c
Saprotroph	18.9 ± 0.91b	22.48 ± 1.67a	12.04 ± 0.41c	16.37 ± 1.88a	12.8 ± 0.10b	10.37 ± 0.06c
Saprotroph-symbiotroph	17.99 ± 0.65a	8.81 ± 0.87b	7.59 ± 0.08c	15.01 ± 1.40a	15.24 ± 0.34a	10.22 ± 0.41c
Symbiotroph	5.65 ± 0.20a	2.1 ± 1.02c	3.06 ± 0.13b	10.3 ± 0.32a	2.55 ± 0.03c	3.88 ± 0.19b
Unassigned	30.24 ± 1.70c	37.59 ± 1.89b	56.36 ± 1.13a	32.59 ± 1.97c	33.26 ± 0.58b	53.27 ± 1.41a

Lower case letters represent significant differences between cultivation methods (*p* < 0.05).

## 4 Discussion

The quality of forest and grass medicinal materials is affected by the medicinal plant species, cultivation methods, ecological conditions, and other factors, and the content of medicinal components reflects the quality of medicinal plants (Yang et al., 2016). Rhizosphere microbial communities are directly or indirectly involved in the growth and development of medicinal plants and the synthesis and accumulation of medicinal components through a variety of pathways (Berendsen et al., 2012). Previous studies have shown that there are significant differences in the main active components of medicinal plants under different cultivation methods (Song et al., 2023). Yang et al. (2024) found that the cultivation method affects the content of medicinal components of *Scutellaria baicalensis*, where cultivated *S. baicalensis* showed significantly higher content of most flavonoids than those of wild populations. In this study, the FP and SC cultivation method significantly enhanced the synthesis and accumulation of the active pharmaceutical components in *E. koreanum*, exceeding the standards of the Chinese Pharmacopoeia. Therefore, appropriate cultivation methods can effectively improve the quality of *E. koreanum*.

Differences in cultivation affect soil physicochemical properties, and microorganisms respond to environmental changes through potential adaptive evolution (Li J. et al., 2024; Wang Z. et al., 2024). Dong et al. (2023) found that the rhizosphere microbial community of wild *Fritillaria pallidiflora* Schrenk tended to be dominated by fungal taxa, whereas the rhizosphere microbial community of cultivated *Fritillaria pallidiflora* Schrenk tended to be dominated by bacterial taxa. In this study, the rhizosphere soil of SC also exhibited higher bacterial diversity, while the rhizosphere community of the bionic cultivated in forest FP tended to include more fungal taxa, which may be related to the relatively similar environment to that of WT under the larch forest. The rhizosphere microbial communities of SC and FP exhibit significant functional advantages. The rhizosphere soil of SC is dominated by phyla such as *Chloroflexi* and *Ascomycota*, which significantly enhance the utilization rate of organic elements by participating in plant nutrient cycling and energy transformation (Challacombe et al., 2019; Narsing Rao et al., 2022). The FP rhizosphere was enriched with *Verrucomicrobiota* and *Patescibacteria*, related to stress tolerance and nutrient cycling, further contributing to plant adaptability to abiotic stresses (Trivedi et al., 2020; Ye et al., 2024). In contrast, the rhizosphere of WT was dominated by *Proteobacteria* and *Basidiomycota*. The reduced abundance of *Basidiomycota* might be related to the inhibitory effect of the cultivation environment on soil microorganisms, consistent with the results of previous studies showing that cultivation reduces the abundance of *Basidiomycota* in *Zea mays* L. (Li Y. et al., 2022). At the genus level, compared with levels in WT, a large number of functional microorganisms related to nutrient absorption and the synthesis of pharmacologically active compounds accumulated in the rhizosphere of SC and FP. For example, microorganisms such as *HSB OF53-F07*, *Bacillus*, and *Humicola* can convert nutrients from ineffective and slow-acting states to effective and fast-acting states, thus enhancing nutrient uptake by plant roots (Lou et al., 2018; Turner et al., 2013). *Gemmatimonas* help plants efficiently utilize soil nutrients by promoting nutrient uptake, which in turn promotes plant growth and development (Zhou et al., 2022); its enrichment in the FP rhizosphere also suggests the recruitment of beneficial microorganisms. In addition, the abundance of rhizosphere *Piloderma* was higher in WT and late post-fruiting nutrient growth of FP than in SC, and the chemicals released from this species may have adverse effects on plants (Li M. et al., 2023), there by influencing the lower levels of key compounds in WT and FP than in SC and reducing the utilization of nutrients by the plant. Enrichment of WT rhizosphere *Amanita* may affect ecological interactions with host plants through the dynamic evolution of toxin genes in the colony, with implications for plant growth and development (Drott et al., 2023). WT rhizospheres showed the aggregation of *Mortierella* (Val-Torregrosa et al., 2022), *Bryobacter* (Liu Z. et al., 2020) and other microbiota associated with plant stress tolerance. In conclusion, different cultivation methods result in differences in the abundance of characteristic rhizosphere microbiota of *E. koreanum*.

Soil properties and enzyme activity levels are important indicators of the natural environment and are highly correlated with microbial activity (Krause et al., 2017; Ma et al., 2021). Bacterial communities in this study were influenced by TK and AP. *Bacillus*, *Humicola*, and *HSB OF53-F07* were positively correlated with TK and AP, and a correlation study on *Dipterocarpus yunnanens* is also showed that the role of AP and AK in shaping bacterial communities (Wang C. et al., 2023). Soil enzyme activities were also related to bacterial communities, and RDA results showed a positive correlation between *Bryobacter* and *Candidatus Udaeobacter* abundance and SSC activity. *Candidatus Udaeobacter* had the highest relative abundance among bacterial genera under different cultivation methods. It can utilize limited carbon sources and participate in the metabolism of amino acids and polysaccharides (Brewer et al., 2016), revealing the role of energy transformation in the association between soil enzyme activity and the microbial community. pH can indirectly and directly regulate bacterial communities. Studies have shown that pH has a significant impact on the aggregation of bacterial communities in tobacco (Jiang et al., 2023; Lammel et al., 2018), consistent with the results of this study. The soil fungal community was less affected by changes in soil pH and had a closer relationship with soil nutrients (Lauber et al., 2008; Rousk et al., 2010), similar to the results of this study. As evaluated using the Mantel test, TN, AN, SOM, and SSC were key factors affecting fungal communities, in line with Wu et al. (2020) and Yang et al. (2019). AN and TN was positively correlated with the abundance of *Piloderma* and *Wilcoxina* and negatively correlated with the abundance of *Humicola*, further demonstrating the impact of nitrogen on specific fungal groups. In addition, the significant associations of *Pseudogymnoascus*, *Tmentella* and other taxa with SSC activity corroborate the regulatory effect of SSC on fungal communities in a study of the rhizosphere microbiota of *Angelica sinensis* (Oliv.) Diels, indicating that enzyme activity may influence fungal functional networks through carbon source metabolic pathways (Xie et al., 2023). These findings suggest that soil properties and enzyme activity levels play important roles in shaping the plant microbiome, in turn influencing the diversity and composition of rhizosphere microorganisms under different cultivation practices.

Ecological conditions are closely related to the growth, development, and quality of forest and grassland medicinal materials (Zhang et al., 2020). There is a significant relationship between the accumulation of pharmacologically active components in *E. koreanum* and rhizosphere microbial community. Studies have shown that Icariin and epimedin content are significantly positively correlated with AK, AP, TK, TP and microorganisms, such as *Bacillus*, *Humicola*, *Gemmatimonas* and negatively correlated with SOM, AN, soil enzyme activities and most fungi. Additionally, higher phosphorus and potassium content and low levels of nitrogen in the soil are conducive to the accumulation of flavonoid components in medicinal plants (Kong et al., 2010; Li Q. et al., 2023; Liu et al., 2021). *Bacillus* is an important component of plant growth-promoting rhizobacteria; in most studies related to medicinal plants, it has been shown to promote the synthesis and accumulation of pharmacologically active compounds (Feng et al., 2021; Guo et al., 2025). *Bacillus* may influence the redox state of the host, helping to overcome critical periods of development and seedling establishment (Pitzschke, 2016), which could also explain why *Bacillus* was more abundant in the full bloom period. *HSB OF53-F07* participates in the metabolism of nutrients in the soil, improves the soil environment, and promotes plant growth (Yang et al., 2021). *Gemmatimonas* not only promotes plant growth but also increases plant tolerance to abiotic stresses (Li P. et al., 2022). *Humicola* can degrade organic molecules in the soil, promote the dissolution of phosphates, increase the availability of soil phosphorus to plants, and enhance the accumulation of active ingredients in medicinal plants (Yang et al., 2014). The growth of most fungi is negatively correlated with the accumulation of active pharmaceutical ingredients in *E. koreanum*. For example, microorganisms such as *Piloderma* and *Wilcoxina*, enriched in the rhizosphere of WT plants, compete for nutrients and inhibit the activity of beneficial microorganisms, thereby affecting plant quality and leading to root rot (Liu Y. et al., 2024; Wang L. et al., 2024). The accumulation of rhizosphere pathogens induces an increase in the abundance of antagonistic rhizosphere pathogens (Bakker et al., 2018); for example, *Mortierella* regulates root rot in plants and induces *P. ginseng* resistance to black spot disease (Ji et al., 2024; Liu L. et al., 2020). Moderate dual-source stress can promote the accumulation of active pharmaceutical ingredients (Zhuang et al., 2023); however, in this study, the synergistic effect of the high nitrogen content in WT soil and pathogens inhibited the accumulation of active pharmaceutical ingredients. These results indicate that differences in soil physicochemical properties and enzyme activity among cultivation methods affect the community composition of rhizosphere microorganisms, which ultimately and indirectly contribute to plant growth and the synthesis of pharmacodynamic components.

Microbial function prediction provides insight into the metabolic functions of the flora (Bell et al., 2023). The functional prediction results of this study showed that the terms membrane transport and lipid metabolism were more abundant in SC and FP than in WT. Membrane transport related functions improve diffusion and transport efficiency, thus enhancing plant adaptation (Martínez et al., 2022). Lipids metabolism are an essential component of microbial biofilms and play a crucial role in the response to environmental stress and survival (Wu et al., 2018). This phenomenon explained the enrichment of nutrient-cycling microorganisms, such as *Bacillus* and *Humicola* in the rhizosphere of SC and FP in this study. Fungal functional prediction indicated that the abundance of saprotrophic fungi was significantly lower in the SC rhizosphere than in WT. Studies have shown that saprotrophic fungi are readily enriched in decaying and diseased plants and are more likely to cause plant diseases (Zhang et al., 2022), further suggesting that the aggregation of pathogenic bacteria, such as *Piloderma*, *Wilcoxina*, in the WT rhizosphere compared with SC and FP may pose an obstacle to root nutrient cycling and the accumulation of key components. The enrichment of pathotroph-saprotroph-symbiotroph fungi resulted in greater resistance and nutrient utilization in the rhizosphere of FP, conferring the potential for *E. koreanum* growth in harsh environments (Shi et al., 2016). The functional prediction results further confirmed the enrichment of the SC rhizosphere for microorganisms related to nutrient accumulation and enrichment of the FP rhizosphere with resistant microorganisms.

From a practical production point of view, although the simulated habitat cultivation samples had higher content of medicinal components, this approach is limited by the smaller number of plots to choose from, large economic investment required, and potential impact on the return of forests, which is not conducive to large-scale production. Therefore, against the backdrop of strengthening the conservation of wild *E. koreanum* resources, the bionic cultivated in forest is preliminarily recommended. During soil management, a “nitrogen control and phosphorus-potassium increase” strategy should be implemented, and the quality of *E. koreanum* can be improved by increasing the abundance of bacterial groups, such as *Bacillus*, *Gemmatimonas* and *Humicola*, in the soil.

## Data Availability

The data presented in the study are deposited in the NCBI repository (https://www.ncbi.nlm.nih.gov/), accession number PRJNA1202879 and PRJNA1202845.

## Footnotes

^1^ www.omicsmart.com
