# Supplementary material for: Differential analysis of the quality and soil microhabitat of Epimedium koreanum Nakai under different cultivation methods
Source: Front Microbiol. 2025 May 22;16:1556173. doi: 10.3389/fmicb.2025.1556173 (PMC12137310; doi:10.3389/fmicb.2025.1556173)
Supplement: Supplementary file 1 [file Supplementary_material.docx]

Supplementary Material

**Supplementary Table 1**. PCR reaction systems (50 μL)

| Q5 Enzyme amplification system | Dosage |
| --- | --- |
| 5x Q5@ Reaction Buffer | 10 μL |
| 5 x Q5@ High GC Enhancer | 10 μL |
| 2.5mM dNTPs | 1.5μL |
| Primer F (10 μM) | 1.5μL |
| Primer R (10 μM) | 1.5 μL |
| Q5@ High-Fidelity DNA Polymerase | 0.2 μL |
| DNA template | 50ng |
| H_2_O | Up to 50 μL |

**Supplementary Table 2.** PCR amplification procedure

| Temperature | Time | cycles |
| --- | --- | --- |
| 95℃ | 5 min |  |
| (95 ℃ | 1 min |  |
| 60℃ | 1 min |  |
| 72℃ | 1 min) | 30 cycles |
| 72℃ | 7 min |  |


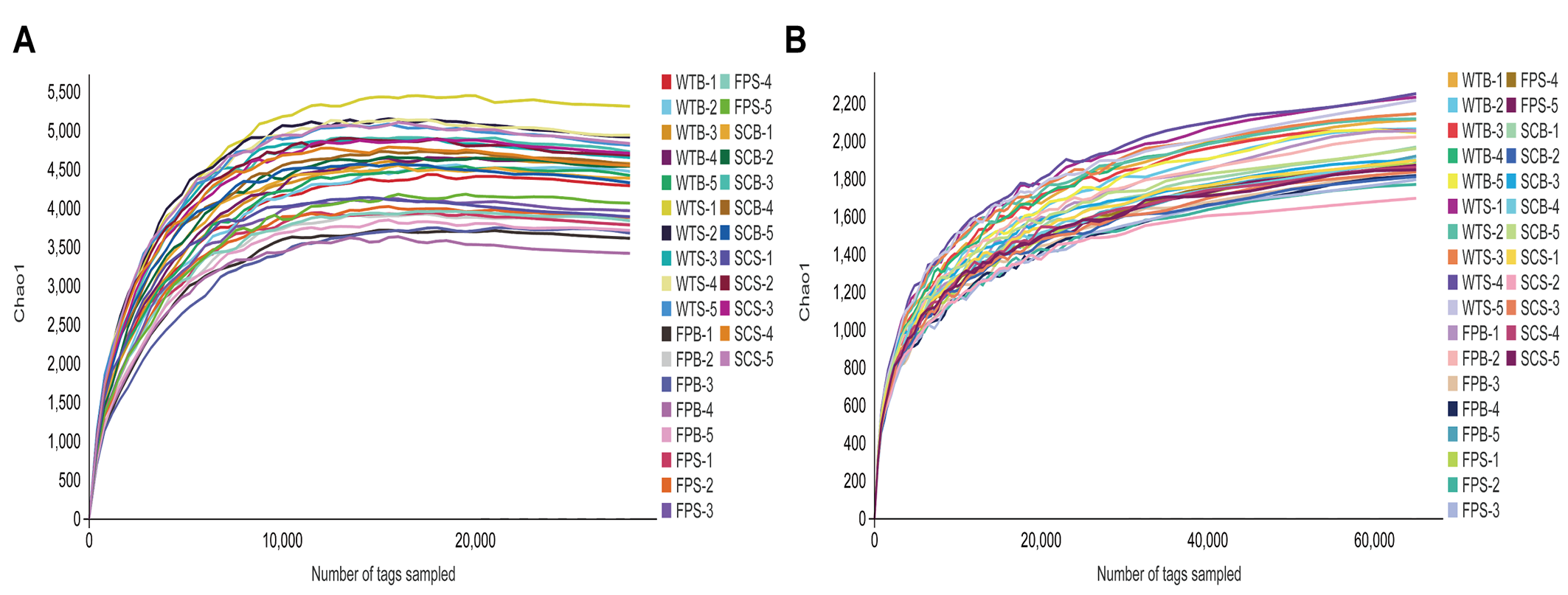


**Supplementary Figure 1.** Dilution curves for soil bacteria (**A**) and fungi (**B**).


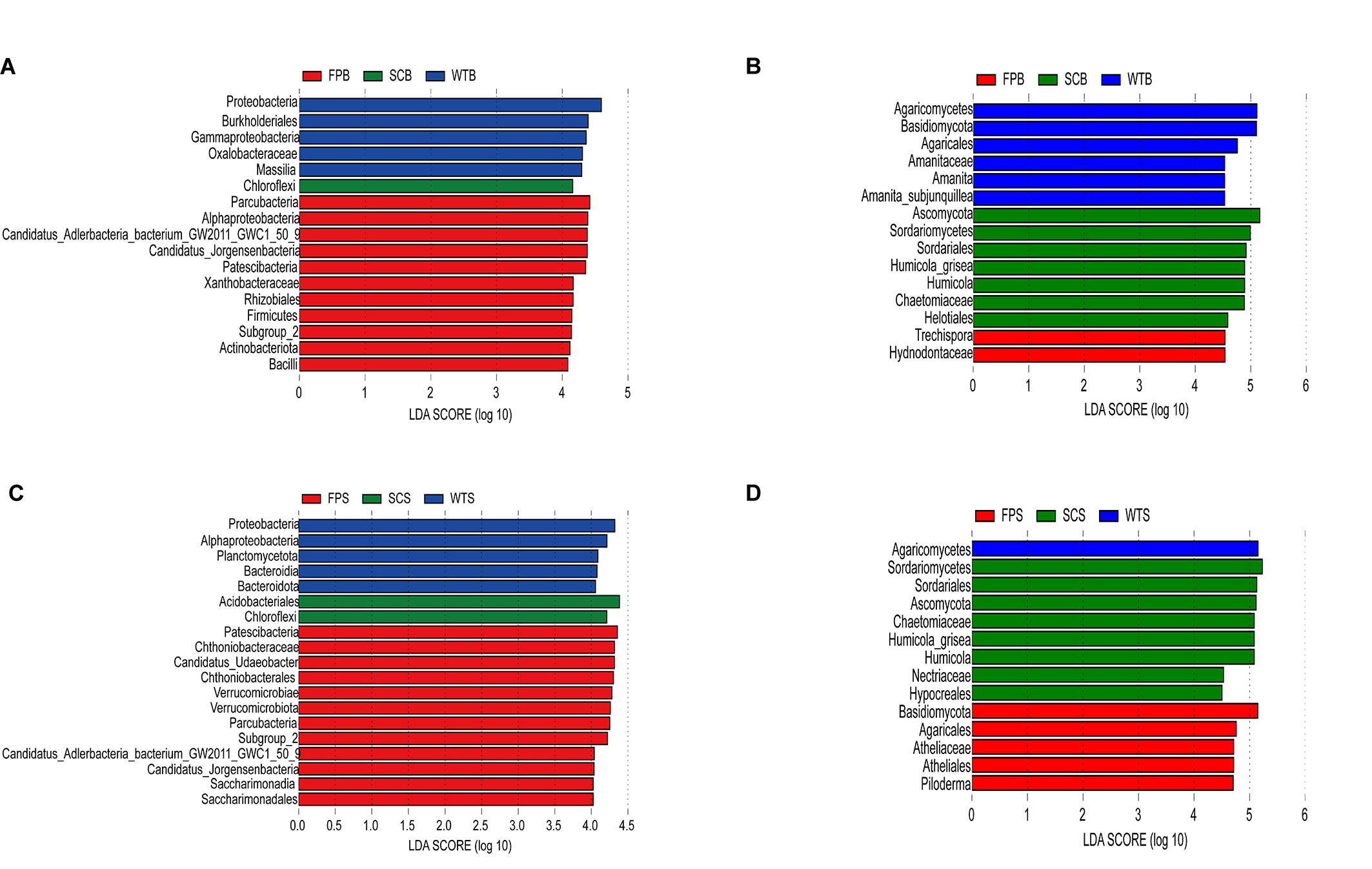


**Supplementary Figure 2.** Histograms of the distribution of LDA values analyzed for soil bacteria (**A,C**) and fungi (**B,D**) indicator species.Bacteria: LDA ≥ 4.0; Fungi: LDA ≥ 4.5.
